# A Simple Method for Developing a Hand‐Drawn Paper‐Based Sensor for Mercury; Using Green Synthesized Silver Nanoparticles and Smartphone as a Hand‐Held‐Device for Colorimetric Assay

**DOI:** 10.1002/gch2.202000099

**Published:** 2021-02-15

**Authors:** Maryamosadat Mavaei, Azam Chahardoli, Ali Fattahi, Alireza Khoshroo

**Affiliations:** ^1^ Pharmaceutical Sciences Research Center Health institute Kermanshah University of Medical Sciences Kermanshah 6715847141 Iran; ^2^ Medical Biology Research Center Kermanshah University of Medical Sciences Kermanshah 6715847141 Iran; ^3^ Present address: Center for Applied NanoBioscience and Medicine College of Medicine Phoenix University of Arizona Phoenix AZ United States

**Keywords:** colorimetric assay, mercury, paper‐based device, smartphone‐based detection, water analysis

## Abstract

Mercury ions are highly toxic at trace levels, and its pollution has posed a significant threat to the environment and public health, where current detection methods mainly require laborious operation and expensive instrumentation. Herein, a simple, cost‐effective, instrument‐free approach for selective detection of Hg^2+^ based on a hand‐drawn paper‐based naked‐eye colorimetric device is developed. To develop a hand‐drawn paper‐based device, a crayon is used to build hydrophobic barriers and a paper puncher is applied to obtain patterns as a sensing zone. A green method for the synthesis of silver nanoparticles (AgNPs) is applied using Achillea Wilhelmsii (Aw) extract. The sensing ability of Aw‐AgNPs toward Hg^2+^ is investigated in both solution‐phase and paper substrate loaded with Aw‐AgNPs using colorimetric methods. For the paper‐based sensor, the quantification of the target relies on the visual readout of a color‐changed sensing zone modified with Aw‐AgNPs. Under optimal conditions, the color of Aw‐AgNPs in aqueous solution and on the coated paper substrate can change from brown to colorless upon addition of target, with a detection limit of 28 × 10^−9^ m and 0.30 × 10^−6^ m, respectively. In conclusion, the present study indicates the potential of this hand‐drawn eco‐friendly paper‐based sensor for monitoring of mercury.

## Graphical abstract

A simple and instrumental‐free paper‐based colorimetric sensor for Hg^2+^ ions is provided based on a smartphone as a hand‐held‐device for colorimetric assays.

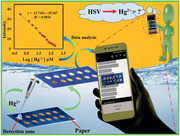

## 1 Introduction

Accompanying the rapid industrialization, a considerable sum of hazardous and deadly contaminants, including the toxic metal ions (TMIs), unscrupulously expel into the environment.^[^ ^1^, ^2^, ^3^, ^4^, ^5^ ^]^ Due to long biological half‐lives, severe toxicity, and their potential bioaccumulation in different vital body organs, contamination by TMIs poses a severe threat and challenge to human health, aquatic life, and the ecological environment.^[^ ^6^, ^7^, ^8^, ^9^, ^10^ ^]^

Mercury (II, Hg^2+^) contamination is the second highly TMIs, with serious health risk factor and as a carcinogenic pollutant to living organisms. Even small doses of bioaccumulated Hg^2+^ can lead to adverse effects and irreversible damages on vital body organs, e.g., brain, spinal cord, with severe dysfunction.^[^ ^11^, ^12^ ^]^ As stipulated by the World Health Organization (WHO), the maximum tolerable limit of mercury ion in the drinking water sample should be around 30 × 10^−9^ m (6 ppb).^[^ ^13^ ^]^ Hence, it is urgent to routinely monitor Hg^2+^ ions as a challenge not only for environmental checking but also for drinking water sources and health protection. The various sophisticated and expensive techniques are available for selective determination of Hg^2+^ ions included atomic absorption spectrometry,^[^ ^14^ ^]^ atomic emission spectrometry,^[^ ^15^ ^]^ cold vapor atomic absorption spectrometry,^[^ ^16^ ^]^ X‐ray fluorescence spectrometry,^[^ ^17^ ^]^ inductive coupled plasma mass spectrometry,^[^ ^18^ ^]^ electrochemical analysis,^[^ ^19^ ^]^ and gas chromatography.^[^ ^20^ ^]^ Although existing detection approaches offer high selectivity and unparalleled sensitivity, most of them have drawbacks of large sample volumes, time‐consuming, bulky instrumentation, and tedious sample preparation procedures that make in situ and on‐site analyses impossible. Compared with mentioned methods, colorimetric sensors with marked advantages, such as visualization, convenient read‐out, rapid analysis, and ease of measurement, are very promising for metal analysis.^[^ ^21^ ^]^ Recently, organic dye colorimetric methods and optical sensors based on size and interparticle distance‐dependent have viewed significant growth in bio/chemosensing and monitoring measures; but, their application got limited by a low color resolution owing to the monocular change‐based signal readout and the intrinsic low extinction ratios of organic dyes probes.^[^ ^22^, ^23^, ^24^, ^25^, ^26^ ^]^ Presently, a variety of highly selective and sensitive nanomaterial‐based colorimetric sensors for the checking of pollutants such as TMIs have been designed, especially from plasmonic‐based metal nanoparticles, owing to their extremely high extinction coefficients and color‐tunable optical properties.^[^ ^27^ ^]^ Metal nanoparticles (i.e., gold and silver nanoparticles) as signal generators and color labels are promising nanomaterials for the design of low‐cost colorimetric probes.^[^ ^28^, ^29^, ^30^, ^31^ ^]^

In recent years, various colorimetric sensors based on AgNPs for the monitoring of TMIs, e.g., mercury ions, have been developed. Despite the multiple advantages of nanomaterial, rigorous reaction conditions, and reducing agents (thiols, NaBH_4_, NaOH) added the cost of purification and chemical raw material during the procedure of fabricating AgNPs.^[^ ^32^ ^]^ Because of this, intense researches have been directed to the development and exploitation of green approaches and efficient synthesis of AgNPs.^[^ ^33^, ^34^, ^35^ ^]^

Although several studies reported colorimetric sensing/monitoring systems of mercury ion based on the colloidal solutions of green synthesized AgNPs, limitation such as low sustainability and difficult transportation made those probes nonsuitable for in situ applications. To this end, few works have studied a stabilized form of the colorimetric sensor on pellet, film, gelatin, or paper, to overcome the drawback caused by the limiting influence.^[^ ^36^, ^37^ ^]^

Paper‐based detection devices open new research avenues to produce inexpensive, simple, single‐use, portable, minimal sample consumption, and rapid analytical strategies for broad application fields, especially for environmental monitoring. Recently many different fabrication techniques of paper‐based sensors such as wax printing, inkjet printing, laser treatment, and photolithographic, which involve enchasing hydrophobic backing materials on the paper substrates, have been reported. Despite providing high fabrication speed and high resolution of the hydrophobic layer, their routine application is limited by the need for both expensive instruments and trained personnel.^[^ ^38^, ^39^ ^]^

Most recently, combining colorimetric probes with paper devices has attracted colossal attention; some facile electronic platforms such as desktop scanner, digital camera, and also smartphone can be used for image collection and data analysis, which significantly reduces the cost and the usage of instrumentation.^[^ ^40^ ^]^ By a combination of a colorimetric paper probe and a smartphone, the instantaneous quantitative monitoring of metal ions is possible.^[^ ^41^ ^]^

With these insights, we aimed to establish simple, low‐cost, and sensitive AgNPs‐incorporated paper‐based sensors using a hand‐drawn paper‐based plate. Patterning of paper substrates was achieved by an equipment‐free, straightforward, rapid, and easy‐handy manner just through a crayon to draw a hydrophobic barrier. Achillea Wilhelmsii‐mediated AgNPs (Aw‐AgNPs) as a green synthesized AgNPs have been used. Detection of Hg^2+^ by Aw‐AgNPs was investigated both in aqueous solution and paper substrate loaded with Aw‐AgNPs using spectrophotometric and colorimetric assays, respectively. Colorimetric analysis was performed using a smartphone application as a portable detector and achieved visual, quantitative detection. Image analysis was performed with HSV (hue saturation value) assessment as a system of color vision. When Hg^2+^ ions are added to the wells of the plate, the color of wells is changed markedly from brown to colorless. The fabricated paper‐based sensing platform has proved to be applicable for the detection and analysis of Hg^2+^ contaminants in various real water samples.

## 2 Experimental Section

### 2.1 Chemicals

All reagents used in the experiment, including sulfuric acid, hydrochloric acid, all metallic and anion salts were purchased from Merck (Germany), which were analytically pure and used in the experiment without further purification. Stock solutions of the anions (1.0 × 10^−3^ m), and metal ions (1 × 10^−3^ m) were prepared freshly in water. Whatman qualitative filter paper grade 3 was purchased from Sigma‐Aldrich, USA.

### 2.2 Synthesis of Silver Nanoparticles

Preparation of Aw‐AgNPs is presented in the Supporting Information.

### 2.3 Instrumentation and Characterizations

The absorption spectrum of Aw‐AgNPs probe was recorded by UV‐visible spectrophotometer (Lambda UV mini‐1240 instrument, Shimadzu, Japon) at a wavelength range of 200–1100 nm using a 10 mm quartz cuvettes. The scanning electron microscopy (SEM) images were performed on an FEI quanta 450.

### 2.4 Spectral and Colorimetric Detection of Hg^2+^

To assessing the colorimetric performances, 22 anionic and cationic salt solutions at the same conditions and with the same concentration (0.5 mL, 10^−3^ mol L^−1^) were added into the diluted solution of fresh prepared Aw‐Ag NPs solution (2 mL, 0.4 mg L^−1^).

### 2.5 Fabrication of Paper‐Based Sensor and Colorimetric Process

**Figure 1:**
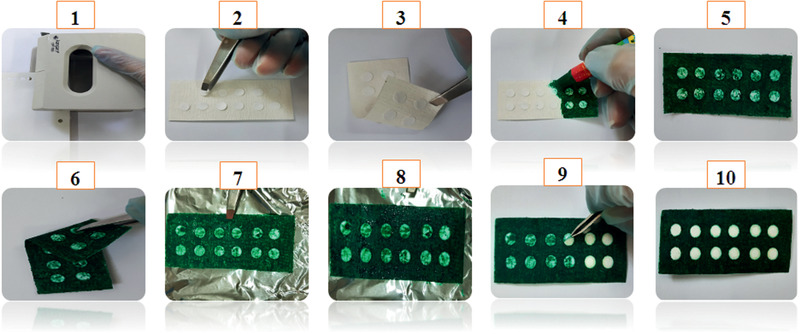
Fabrication procedure of paper‐based sensor.

First, the paper substrates were fabricated by a manual and straightforward manner using a circular stencil as well as a crayon.^[^ ^42^ ^]^ First, the filter paper was cut in 3 × 7 cm^2^ before use. The sensing zones were covered by the circular stickers prepared from self‐adhesive glossy photo paper using a paper puncher; for this purpose, the circular stickers were placed on double side of the filter paper. Then, both sides of the filter paper were drawn using a crayon. The patterned paper was then placed on a hotplate at different temperatures and time ranges to optimize the melting and penetration of crayon to the filter paper for forming hydrophobic barriers. Then, the circular stickers of the top layer were detached; the production approaches of the paper‐based sensor are illustrated in **Figure** **1**. For optimizing the process conditions, the effects of melting temperature, time for providing a paper‐based plate, amount of Aw‐AgNPs and Hg^2+^ ions on detecting zone, and response time were evaluated.

Typically, 10 µL of Aw‐AgNPs was cast on the test zone of each paper and dried at room temperature. Subsequently, 20 µL of various concentrations of Hg^2+^ was loaded to each test zone of paper and kept at room temperature and different times to optimize the contact time between AW‐AgNPs and Hg^2+^ ions. The images of the paper‐based sensors were captured using a Samsung Galaxy A50, and the intensity of their colors of images was analyzed by the color picker as a program implemented on a smartphone. The concentration of Hg^2+^ ions was proportionate to the different intensities values.

### 2.6 Samples Information and Sampling Sites

The water samples were collected (Date: 10th August 2019) from the Gharasu river basin, Sarab Ravansar, and Sarab Qanbar lakes in Kermanshah Province (west of Iran). These regions are located in longitudes 47°03′60.00″ E and latitudes 34°17′60.00″ N. The surface and groundwater in these regions supply the agriculture water as well as the drinking water, so an appraisal of water was quite necessary for these zones. All samples were collected by spot sampling technique and were stored in the water sampling bottles. Afterward, these samples were acidified about pH ∼ 2 using sulfuric acid 50% v/v based on the APHA standard measurement method. At last, the water samples were kept and transferred to the research laboratory for further testing. Before the colorimetric process, the pH was changed to about 6–6.5.

### 2.7 Real Water Samples Tests

The applicability of our colorimetric method to detect Hg^2+^ contaminants in real water samples, from well, river, and lake water, was investigated. For this purpose, several concentrations of Hg^2+^ were spiked to the collected water samples and sensed with the colorimetric probe either in solution or designed paper‐based sensor; the change of intensity was recorded by a UV‐visible spectrometer and the smartphone application, respectively. Standard deviation was also achieved through the data average of the three independent tests.

## 3 Results and Discussion

### 3.1 Synthesis Procedures and Characterization of Aw‐AgNPs

In recent past, several metal NPs‐based colorimetric probes have been attracted attention and designed for Hg^2+^ monitoring by modifying the surface of nanoparticles with analyte‐specific ligands. Although these approaches exhibit good responses for target analytes, most of these ligands are still defective because of high cost, low water solubility, the unsteady structure, time‐consuming analysis, complicated synthesis, and modification of sensing probes. Besides, most of the approaches used to produce NPs are not green. Despite the many advantages of NPs, most of them possess toxicity problems and have high potential risks for human and environmental health. Therefore, the use of renewable bionanomaterials and the improvement of green strategies for the synthesis of NPs have gained worldwide attention recently. In particular, AgNPs as reporting probe for the colorimetric detection and visual sensing of Hg^2+^ ions have been invested. In this study, we used a green sustainable Aw‐AgNPs as nanoprobe (Figure S1, Supporting Information). Details on synthesis and characterization of NPs are presented in the Supporting Information.

### 3.2 Fabrication of Paper‐Based Sensing Platform

To further ease the monitoring strategy and form it field deployable, we tried to design and fabricate a paper‐based sensor by using Aw‐AgNPs as a probe layer on a paper. The paper has several features and advantages such as abundant, ubiquity, biocompatible, accessibility, and inexpensive; it operates mainly by capillary force without the assistance of external pumping. It has some particular features like scalable, disposable, and simply modified, which make it more suitable for sensor application. Compared with simple test strips like litmus paper, paper‐based sensors now mainly rely on the fabrication of paper testing substrates (e.g., hydrophobic treatment) for improving their performance and functionality. The fabrication of paper testing sensors can be achieved via various hydrophobic treatments.^[^ ^43^, ^44^, ^45^, ^46^ ^]^ In our experiment, we made the paper plate through a manual and straightforward method by using a crayon and stencil (Figure 1). The application of crayon as a hydrophobic barrier to confine the sensing regions, and stencil as a coat to keep the hydrophilic sensing regions was the principal strategies in the present study. Crayon is easy to obtain and avoids using organic solvents, but less control on the penetration of the melted crayon into paper substrates by capillarity force, results in ill‐defined patterns and also resolution‐decreased patterns, which can be solved by adding a post heating step at a definite time and temperature. Designing paper substrates was carried out by easy, handy, and cost‐effective manner just by using a crayon to draw hydrophobic barrier on double side. Interestingly, the top hydrophobic layers could cling tightly to the bottom hydrophobic layers after the diffusion of the crayon solution from top layers to the bottom layers due to the penetration effect. Thus, the self‐adhesion of the two hydrophobic layers can generate a good barrier.^[^ ^38^ ^]^ Hand‐drawing process has advantages of ease to use and transport in the environmental applications. For this purpose, we offered a straightforward way for patterning paper substrates as a platform for an economical, low‐volume, and portable assay. Painting paper with crayon provides remarkable advantages, which are inexpensive and accessible to use. It is noted that in the whole process, just two steps are needed (painting paper and heating). Generally, it takes ≈5–15 min to perform the procedure. All used materials consisted of a ruler, a crayon, and a heating system are accessible and low‐cost.

### 3.3 Optimization of Experimental Parameters

For providing a paper sensing device with the best performance, the effects of the following parameters were also optimized: a) melting temperature and time for providing a paper‐based plate, b) amount of Aw‐AgNPs, and Hg^2+^ ions on detecting zone, c) response time. The following experimental conditions were found to give the best results: a) melting temperature and time: 70 °C and 30 s; b) best amount of Aw‐AgNPs and Hg^2+^ ions solution: 10 and 20 µL, respectively; c) optimal response time: 1–20 min (Figure 1).

**Figure 2:**
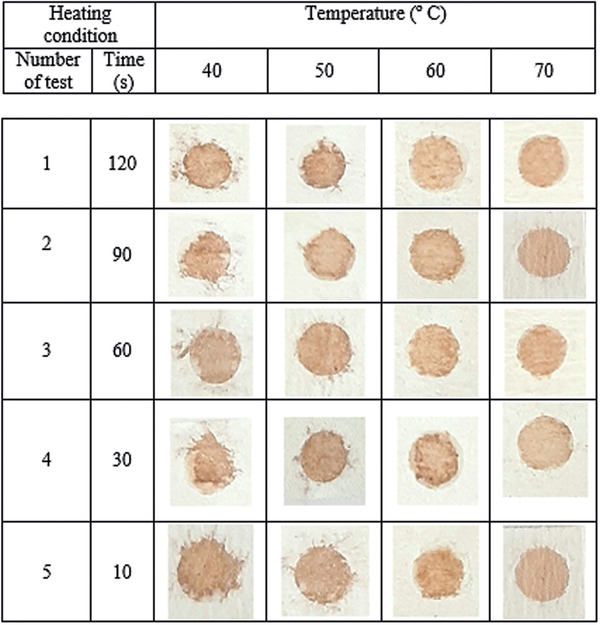
Influences of heating time and temperature on crayon diffusion and the formation of the detecting zone.

Adequate melting time and temperature for crayon diffusion were studied, to identify the optimum temperature required for crayon diffusion throughout the depth of the paper testing substrate. A hydrophobic barrier was drawn on both sides of the laboratory paper testing substrate using a crayon. The paper substrates were then heated on a hotplate for different time and temperature. The representative image of paper fabrication is shown in **Figure** **2**. It can be seen from this image that the crayon diffusion and the reproducible formation of the detecting zone depend on the melting temperature and melting time. It is essential to study the influence of these two factors on the detecting zone in the paper. Therefore, the Aw‐AgNPs are cast onto a sensing zone to consider the distribution and migration of the NPs. As shown in Figure 2, the sensing zone can sufficiently contain the brown‐colored Aw‐AgNPs within the hydrophilic zone if the paper testing substrate is heated at 70 °C. At 40, 50, and 60 °C, the crayon fails to diffuse throughout the depth of the testing paper entirely, but, in 70 °C the melted crayon penetrated the thickness of the test paper to create a hydrophobic barrier on the paper. In the next step, the different melting times of the crayon (between 10 and 120 s) were evaluated. 30 s is a suitable time for crayon diffusion to provide the normal sensing zone and the creation of hydrophobic barriers on paper testing substrates.

The volume of Aw‐AgNPs and Hg^2+^ ion solution is also another factor that was considered in a range of 5–30 µL. The sensing zone immobilizes completely when the amount of Aw‐AgNPs (0.5 mg L^−1^) was 10 µL, and the amount of Hg^2+^ ions solution sufficiently required to cover is 20 µL. Thus, the optimum amounts of Aw‐AgNPs and Hg^2+^ ion solutions were 10 and 20 µL, respectively.

**Figure 3:**
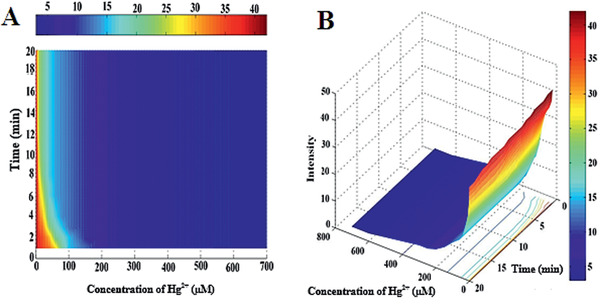
Dependence of reaction time of Aw‐AgNPs with Hg^2+^ on response curve (intensity vs time and concentration of Hg^2+^). Here, the time range was 1 to 20 min, and the concentration range of Hg^2+^ ions was 1 × 10^−6^ –700 × 10^−6^ m; A) 2D and B) 3D plot.

The response time of a sensor is a crucial variable for the application of colorimetric and naked eye recognition assay in laboratories or commercial utilization. We investigated the reaction time of the Aw‐AgNPs at the surface of paper and solution at different times (1–20 min) to detect Hg^2+^ ions. Rapid response time of less than 5.0 min was observed for the Aw‐AgNPs probe in 1 × 10^−6^ m solution of Hg^2+^ ion, which stayed unchanged in the next 20 min (Figure S2, Supporting Information). The reaction time of Aw‐AgNPs on the surface of the paper with different concentrations of Hg^2+^ ions (1 × 10^−6^–700 × 10^−6^ m) was also investigated. **Figure** **3** shows the dependence of the reaction time of Aw‐AgNPs with Hg^2+^ on the response curve (intensity vs time and concentration of Hg^2+^); it can be observed from this figure that the intensity value depends on the concentration of Hg^2+^ and reaction time of Aw‐AgNPs with Hg^2+^. When the low concentrations of Hg^2+^ were utilized, the reaction time of 5 min was not enough. A possible reason for this phenomenon owes to the heterogenous phase reaction and besides a decrease of concentration of target on the paper testing substrate. The reaction and color change was well completed when the reaction time increased to 15 min. Moreover, with increasing contact time or reaction time, (more than 15 min), intensity stayed unchanged.

### 3.4 Surface Characterization of the Paper Sensor

**Figure 4:**
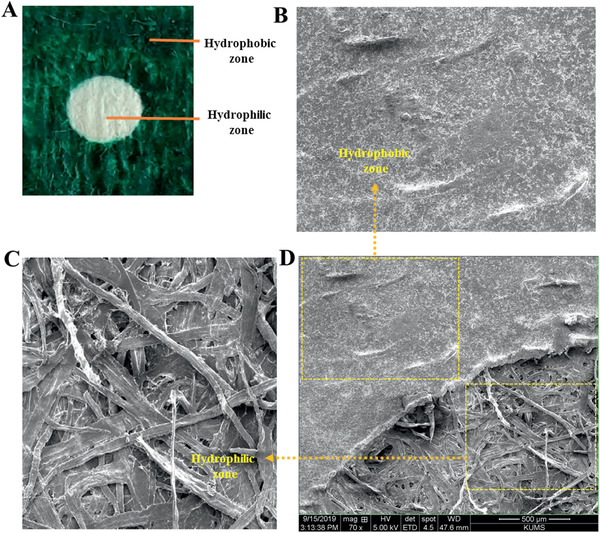
A) Photo and B–D) SEM images of a typical test paper. Strips (B), (C), and (D) represent the structures of the pure filter papers (for the hydrophilic zone), crayon‐covered paper (for the hydrophobic zone), and hydrophobic/hydrophilic interface.

The influence of crayon treatment onto the morphology of paper‐plate was evaluated. The SEM study of different zones showed that the hydrophobic zone and hydrophilic zone had been well made on the paper, and the hydrophobic barrier layer on paper was sufficient enough to block or reduce the interspace of paper testing, as shown in SEM images (**Figure** **4**). It was evident that the paper plate was a highly porous network. After immobilization of crayon into the paper surface, the pores of the paper were almost accumulated and saturated, except in the sensing zone.

### 3.5 Sensing Mechanism of Hg^2+^

AgNPs are promising nanomaterials for the design of low‐cost colorimetric probes; their localized surface plasmon resonance (LSPR) properties have been used in LSPR‐based sensing. LSPR property is chiefly related to the shape, composition, size, surface nature, the dielectric constant of the system, and interparticle distance. LSPR peak and also the color of the systems may be easily changed with establishing a small variation in these factors. Chemical surface reactions like amalgamation and aggregations lead to noticeable changes in color and optical signals.^[^ ^47^ ^]^ In aggregation‐based LSPR colorimetric sensing approaches, a reduction in the spacing between particles affects the plasmon fields of the nearby particles and causes overlapping between them. Consequently, this phenomenon induces a red‐shift in the LSPR peak(s), increasing the intensity and changing the position band.^[^ ^48^ ^]^ The spectral outcome of this study displayed a blue‐shift and a reduction in the LSPR absorption spectrum by adding the Aw‐AgNPs probe solution to Hg ions. It was shown that the LSPR peak of Aw‐AgNPs probe reduced and formed Ag‐Hg amalgam.^[^ ^49^ ^]^ Hence, the blue‐shift of the LSPR peak position confirmed the deposition of Hg on the surface of AgNP; redox interaction between the divalent of mercury (Hg(II)/Hg(0)) and zero‐valent of silver (Ag(I)/Ag(0)) caused a shell layer of Ag‐Hg amalgam on the AgNPs.^[^ ^50^ ^]^ The atomic layers of Hg wrapped around the AgNPs and formed Ag‐Hg and observed a decline in plasmon peak.^[^ ^51^, ^52^ ^]^ Meanwhile, the redox activity could be associated with the different reduction potentials of Hg and Ag. Since the standard reduction potential of Hg [Hg(II)/Hg(0)] (0.85 V) is higher than that of Ag ions [Ag(I)/Ag(0)], (0.80 V), Hg ^2+^ can be able to oxidize Ag particles spontaneously with the redox reaction and also form Hg^0^ layer and amalgam structures.^[^ ^53^ ^]^

### 3.6 Evaluation of the Salt Effect

Colloidal stability is influenced by different environmental factors surroundings such as ionic strength, acidity, and electrolyte composition. Therefore, we controlled Aw‐AgNP stability under three conditions. The impact of ionic strength on the LSPR peak was examined by altering the sodium chloride (NaCl) concentration from 1 × 10^−3^ to 100 × 10^−6^ m. No distinct change in LSPR peak takes place in the presence of NaCl, hinting that the prepared Aw‐AgNPs probe is stable for application in complex environments. The effect of salt on the generation of amalgam was studied, as well; after adding the Aw‐AgNPs to the vial containing 3 mL of Hg^2+^ solution, 1 mL of 1 m NaCl was added into each vial. No AgCl precipitate or aggregate was existed in the system, thus approving that the amalgamation happened. Certainly, this process competes with the adsorption of chloride ion and the form of AgCl. The effect of acidity and electrolyte composition by adding the HCl and phosphate saline buffer (PBS) were tested, respectively. In all cases, no turbidity or precipitation and aggregation were observed. It shows the Aw‐AgNPs was stable without shifting the LSPR band after 8 h.

### 3.7 Smartphone Application for Quantitative Assay

Recently, smartphones, as a pocket lab, can reach their powerful and varied functions by installing various types of programs presented by third‐party service providers. Therefore, they have been extensively utilized in different fields for in situ monitoring.^[^ ^54^ ^]^ Despite the advancement of smartphones, the presence of variables, e.g., ambient light and temperature, and the lack of control over experimental factors similar to what happens in the laboratory affect the sensitivity and accuracy of the colorimetric sensors. Moreover, photoanalysis is not always straightforward, especially when the changes in color are small. In this case, the use of the red, green, blue (RGB) values maybe not possible, but as a substitute to analysis, HSV spaces may be used. Hence, due to the increase of Hg^2+^ ion concentration, the colorimetric probe shows continuous color changes from brown to colorless, which can be used as the premise for the evaluation of the Hg^2+^ ions color picker app.

### 3.8 Analytical Performance of the Paper‐Based Hg^2+^ Assay

#### 3.8.1 Interference Study

**Figure 5:**
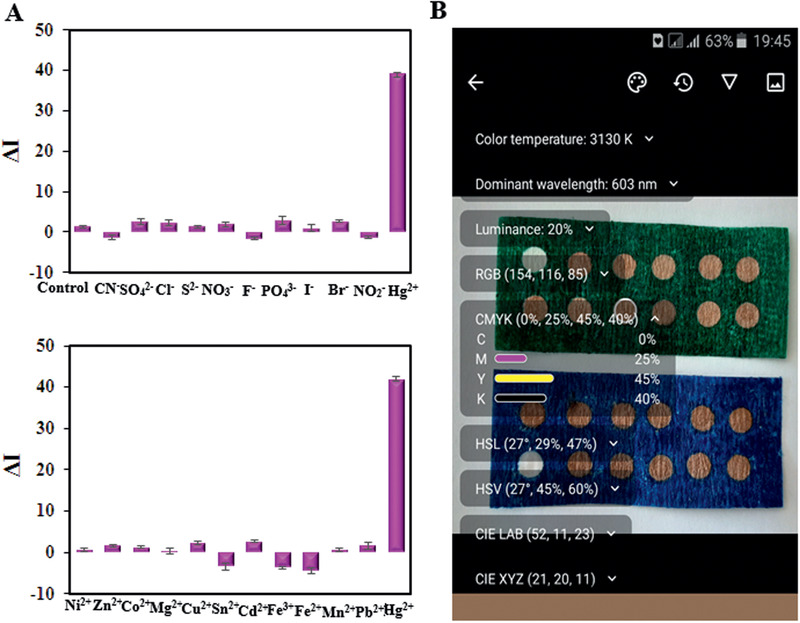
A) The selectivity observed for paper test sensors toward Hg^2+^ in comparison with various metallic ions (blue stripe) and anion ions (green stipe); B) Selectivity observed for paper test and analyzed by the smartphone. Inset: the digital photographic images.

Selectivity is a main challenge of the colorimetric sensor as this feature determines its efficiency to sense the target analyte in the presence of other interfering ions. To investigate the selectivity test of the proposed platform toward Hg^2+^ ion, its colorimetric performance was studied in the presence of different interfering ions such Fe^3+^, Fe^2+^, Mn^2+^, Cu^2+^, Ni^2+^, Hg^2+^, Co^2+^, Cd^2+^, Pb^2+^, Mg^2+^, Zn^2+^, Sn^2+^, PO_4_^3−^, SO_4_^2−^, S^2−^, NO_3_^−^, NO_2_^−^, F^−^, I^−^, CN^−^, Cl^−^, and Br^−^ at the concentration with a fivefold excess of the primary ion, Hg^2+^ (100 × 10^−6^ m). The various ions (metal and anion ions) were examined separately in the existence of Hg^2+^ by the Aw‐AgNPs on the surface of paper and solution probe, provided the selective response to Hg^2+^ (**Figure** **5** and Figures S3 and S4, Supporting Information) successfully. As viewed, only the addition of Hg^2+^ can turn the Aw‐AgNPs platform colorless. This change in color can be ascribed to the sensing process that includes three stages: 1) the electrostatic interaction between the nanoprobe and Hg^2+^ ion, by assisting the reduction of Hg^2+^ ions to Hg atoms; 2) adsorption of Hg atoms on the nanoparticles and diffusion of Hg atoms into the nanoparticles; 3) gradual formation of amalgam structure with the further diffusion of the Hg atoms.^[^ ^55^ ^]^ Also, the error bars in Figure 5 are low, which shows that the accuracy and reproducibility of the analysis are acceptable.

#### 3.8.2 Qualitative and Quantitative Assay

The term “sensitivity” is employed to explain analytical performance. In this sense, the analytic sensitivity is typically defined by a calibration curve with sufficient accuracy or as a change in response signal obtained per unit concentration of target analyte. For the evaluation of Aw‐AgNPs as a colorimetric probe of Hg^2+^ ions, the LSPR sensitivity of the Aw‐AgNPs probe was measured by plotting the changes in the response signal intensity versus various concentrations of Hg^2+^ ions (Figure S5, Supporting Information). A good linear relationship between the Hg^2+^ ions concentration level and sensor response was obtained in ranges of 100 × 10^−9^ to 100 × 10^−6^ m of the Hg^2+^ concentration, and the limit of detection was 28 × 10^−9^ m. Generally, most of the reports include the AgNPs in the solution phases for Hg^2+^ sensing. There have been limited attempts to develop inexpensive AgNPs‐based colorimetric sensors in the solid phase. Kalam et al. prepared the green synthesis of AgNPs and utilized the paper strip as the solid substrate for qualitative sensing of different metal ions, that spherical AgNPs are incredibly selective to Hg^2+^ detection with LOD 1.06 × 10^−6^ m. No data regarding the real field analysis on regular drinking water were reported.^[^ ^56^ ^]^ Also, Ismail's group proposed a paper‐based AgNPs probe for the recognition of Hg (II), Cr (VI), and ammonia in solution. They found a linear detection range of 50 × 10^−3^ to 50 × 10^−6^ m for Hg^2+^ ion. At lower concentrations of Hg^2+^, the reaction time of AgNPs with target took more than 1 h, and no data regarding the real field analysis on regular drinking water were presented.^[^ ^57^ ^]^ The results of our work proved that Aw‐AgNPs exhibited specific selectivity for Hg^2+^ ions in the presence of various ions. Therefore, the rational design of the paper‐based device with low cost, high selectivity, rapid analysis, and high stability, is very desired and suitable for promoting the analysis system.

**Figure 6:**
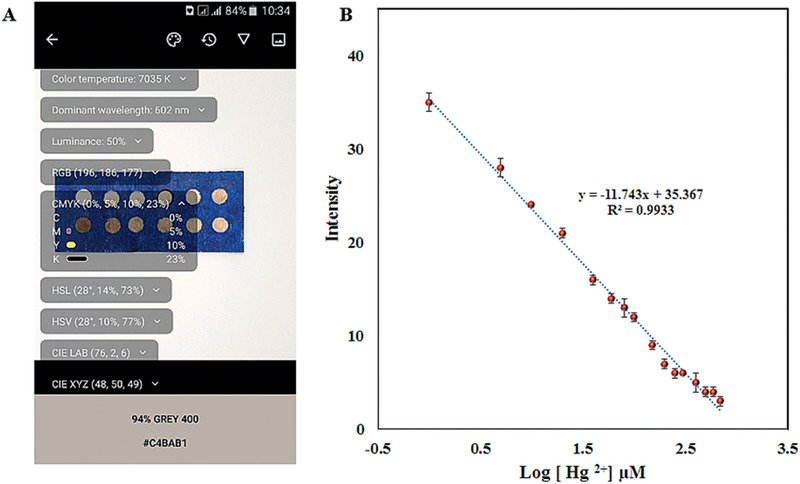
A) HSV photographs of colorimetric responses of the paper sensor toward different concentrations Hg^2+^ ions. B) Concentration‐dependent colorimetric signal readout displayed on a logarithmic scale.

Under the optimized conditions, the hand‐drawn fabricated paper‐based Aw‐AgNPs were used for the one‐step detection of Hg^2+^ in a series (1 × 10^−6^–700 × 10^−6^ m) of Hg^2+^ solutions. The brown color of Aw‐AgNPs gradually faded out in the sensing zones from 1 × 10^−6^ m onward, and the color vanished at 60 × 10^−6^ m. The intensity of the color proportionally decreased with increasing the target concentration. Even by naked eye, we could be able to monitor the sensing system. The digital photos were imaged with a smartphone and data processed using the HSV color system via color picker (**Figure** **6**A). Figure 6B presents a calibration curve between the Hg^2+^ concentrations from 1 × 10^−6^–700 × 10^−6^ m and the color intensity. The linear regression equation was *I* = −11.743 C + 35.367, where *I* was the response to color change or color intensity, and the LOD was 0.3 × 10^−6^ m. The increase in detection limit in the paper system compared solution system could be due to the decrease of interaction between AW‐AgNPs and Hg^2+^ ions.

#### 3.8.3 Method Validation

**Figure 7:**
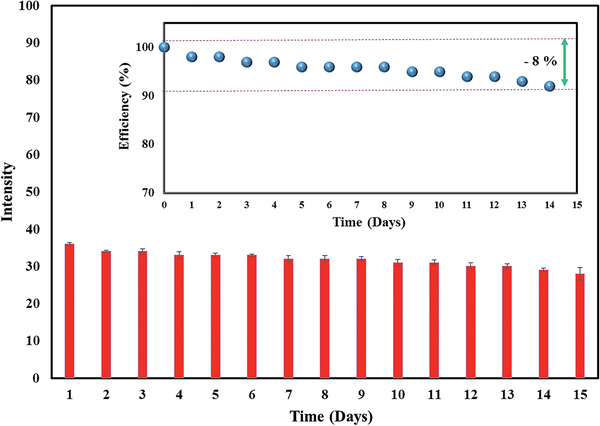
Data plot on intraday investigation viewing the change of intensity on designed paper test using Hg^2+^ solution (1 × 10^−6^ m). The changes in the signal readout of the colorimetric paper‐based are represented as percent efficiency (inset).

The stability of the colorimetric sensor in long‐term performance was investigated for 2 weeks. **Figure** **7** illustrates that the signal intensities in the sensing zones were not deterred over time, which indicates inertness of hydrophobic barrier and nanosensor in the paper‐based platform. Based on changes in signal intensities, we approximated the variation in the efficiency of the suggested paper‐based sensor for Hg^2+^ ions sensing (Figure 7, inset). At the end of 2 weeks, the effectiveness of the suggested paper‐based Aw‐AgNPs was decreased to ≈92%, showing a reliable change (−8%). This amount for the first 7 days was 96% and then reduced to 92% in the next 7 days.

### 3.9 Analysis of Practical Samples based on the Proposed Paper Assay

Not only the selectivity of the proposed probe for detecting mercury ions compared with 22 various interferences ions was verified, but also its applicability in the actual water samples was evaluated in laboratory conditions. The practicability of colorimetric test papers was investigated by using the river, well, and lake water. Hg^2+^ solutions with various concentration levels were spiked to the collected environmental water samples. A drop of Hg^2+^ solution on the surface of the paper caused the eye‐readable color change, from brown to colorless; this trend of color changes shows that the designed paper sensor can provide the affordable visual sensing for Hg^2+^ in real water samples. Then, we acquired HSV spaces of the change color of the sensing zone with the smartphone app. In the river, well, and lake water, the recovery rates calculated by HSV spaces were, respectively, obtained to be 84−113.31%. The recovery factor illustrates that the color changing on the sensing zone of the test paper monitored by the smartphone app is valid for the quantitative sensing of Hg^2+^ ions (**Table** **1**). Furthermore, the practical application of Aw‐AgNPs in solution was investigated. Based on the obtained results in Table S1 in the Supporting Information, the recoveries for water samples were found to be in the range of 90.5–110.5%. The results illustrated a definite agreement between the obtained and certified values. Thus, the presented Aw‐AgNPs probe has great application potential for monitoring Hg^2+^ in real water samples. The sensing platform offers a higher accuracy compared with cross‐references and is capable of detection and discrimination of Hg^2+^ in even real water samples. It shows excellent potential for qualitative and also quantitative analysis with a sensitivity below the value safe limit concentrations (30 × 10^−9^ m) and a controlled error range.

**Table 1:** Probing of Hg^2+^ ion in actual water samples (river, lake, and well water) spiked with various concentrations of Hg^2+^ ion by the designed paper sensor

Sample	Amount added [× 10^−6^ m]	Amount found [× 10^−6^ m]	Recovery [%]	RSD [*n* = 3, %]
Well water				
1	20	20.5	102.5	2.45
2	50	51.5	103	1.14
3	100	113.31	113.31	4.28
River water				
1	20	16.8	84	0.57
2	50	45.9	91.8	4.1
3	100	96.4	96.4	9.64
Lake water				
1	20	17.7	88.5	3.52
2	50	50.7	101.4	0.48
3	100	89.10	89.1	2.72

## 4 Conclusion

In this study, we introduced a smartphone‐assisted colorimetric paper‐based nanosensor that has the advantages of convenient fabrication, reliable sensing, easy handling, high stability, and sensitive detection of Hg^2+^ in both solution and paper‐based sensor; the design of paper could be achieved by crayon, with the merits of cost‐efficient, and accessible‐to‐use, and silver nanoparticle are from a green source. The whole sensing process can be completed within a portable paper and pervasive smartphone, and the color HSV spaces of the paper‐based Aw‐AgNPs can be recognized by the smartphone, which developed an instrument‐free, and simple on‐site readable analysis. Notably, the colorimetric paper‐based analytical assay based on a smartphone application can introduce quantitative and visual sensing/monitoring of Hg^2+^ ions in environmental water samples. Therefore, a combination of the suggested paper‐based analytical platform with a smartphone app may render the potential toward the subtle sensing of practical environmental water analysis, which can further develop on‐site actual sample monitoring.

## Conflict of Interest

The authors declare no conflict of interest.

## Supporting information

Supporting Information

## Data Availability

Data are not shared.
