# Supplementary material for: A Simple Method for Developing a Hand‐Drawn Paper‐Based Sensor for Mercury; Using Green Synthesized Silver Nanoparticles and Smartphone as a Hand‐Held‐Device for Colorimetric Assay
Source: Glob Chall. 2021 Feb 15;5(4):2000099. doi: 10.1002/gch2.202000099 (PMC8025396; doi:10.1002/gch2.202000099)
Supplement: Supplementary file 1 — Supporting Information [file GCH2-5-2000099-s001.pdf]

© 2021 Wiley-VCH GmbH

Global Challenges Open Access

Supporting Information

for *Global Challenges,* DOI: 10.1002/gch2.202000099

A Simple Method for Developing a Hand-Drawn Paper-Based Sensor for Mercury; Using Green Synthesized Silver Nanoparticles and Smartphone as a Hand-Held-Device for Colorimetric Assay

Maryamosadat Mavaei, Azam Chahardoli, Ali Fattahi, and Alireza Khoshroo**

A Simple Method for Developing a Hand-Drawn Paper-Based Sensor for Mercury; using Green Synthesized Silver Nanoparticles and Smartphone as a Hand-Held-Device for Colorimetric Assay

Maryamosadat Mavaei [a], Dr. Azam Chahardoli [a], Dr. Ali Fattahi[a,b,*], Dr. Alireza Khoshroo [a,*]

[a] *Pharmaceutical Sciences Research Center, Health institute, Kermanshah University of Medical Sciences, Kermanshah, Iran*

[b] *Medical Biology Research Center, Kermanshah University of Medical Sciences, Kermanshah, Iran*

[*] Corresponding authors:

Ali Fattahi:

E-mail: a.fatahi.a@gmail.com

Alireza Khoshroo

E-mail: khoshroo.a.r@gmail.com

ORCID iD: http://orcid.org/0000-0001-5890-0579

Synthesis of Aw-AgNPs

Aw-AgNPs was prepared by green synthesis method based on *Achillea wilhelmsii* C. Koch (Aw) aqueous extract and the details of synthesis and characterization submitted as an independent manuscript [1]. Briefly, 0.015 g of silver nitrate was dissolved in 90 mL deionized water and 10 mL of Aw aqueous extract was added to the above solution, then mixture was exposed to sunlight irradiation. The final product was centrifuged (10000 rpm, 30 min) and washed repeatedly with deionized water to obtain Aw-AgNPs.

Characterization of Aw-AgNPs

The absorption spectrum of Aw-AgNPs was recorded by UV–visible spectrophotometer (Lambda UV mini-1240 instrument, Shimadzu, Japon) at a wavelength range of 200 to 1100 nm using a 10 mm quartz cuvettes. Figure 1SA shows the UV-vis spectrum, the peak observed at 440 nm absorption band corresponding to Aw-AgNPs**.**

The size and morphology of AgNPs were determined using a transmission electron microscope (TEM), (LEO 906 microscope, Carl Zeiss, Germany). In TEM analysis, some structural features such as morphology, particle size, etc. are shown given in Fig. 1SB. The mono-dispersed Aw-AgNPs are spherical in shape, and the particle size ranged between 4-42 nm.

The Crystalline structure of AgNPs was confirmed by an X-ray diffraction (XRD analysis) (APD 2000 X-ray generator, Novara, Italy, at a voltage of 40 kV and a current of 30 mA with Cu K-alpha radiation). Based on XRD spectrum, four distinct diffraction peaks of Aw-AgNPs was indicated at 38.15º, 45.01º, 64.5º and 77.45º, which indexed at 111, 200, 220 and as well as 311 planes respectively [2]. The face-centered cubic (FCC) structure of Aw-AgNPs and their crystalline nature image was approved by XRD (Fig. 1SD). In addition, the crystallite sizes of

Aw-AgNPs using the Debye-Scherer equation and by taking into account the (111) Bragg reflection was 10.8 nm.

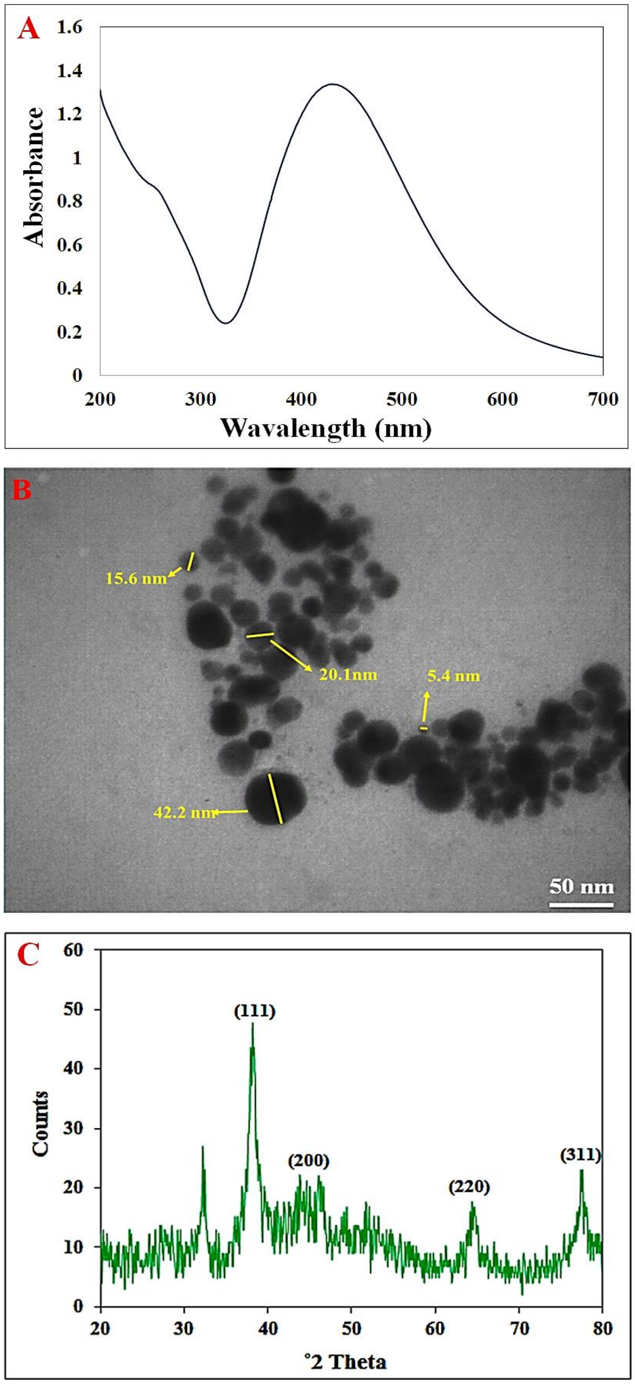

Figure S1: UV–Vis spectrum of Aw-AgNPs (A); TEM images of synthesized Aw-AgNPs (B); XRD pattern of biogenic Aw-AgNPs (C).

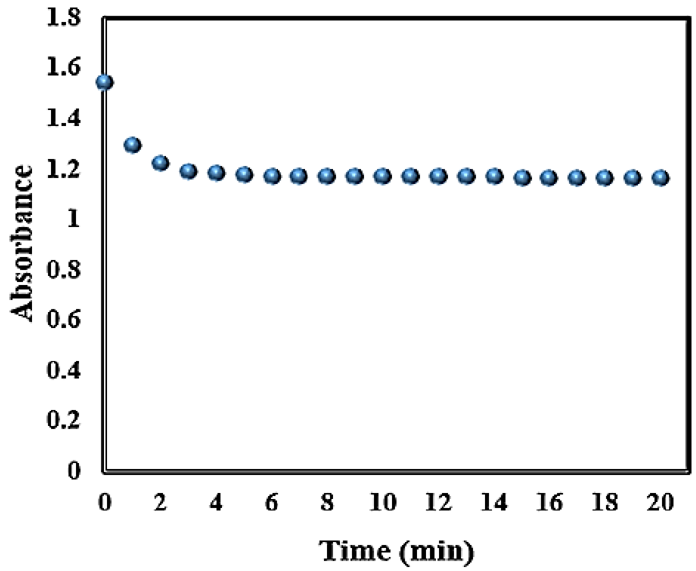

Figure S2: The time response plot of the prepared Aw– AgNPs nanosensor at 440 nm in the presence of $[Hg^{2+}]$ = 1 μM.

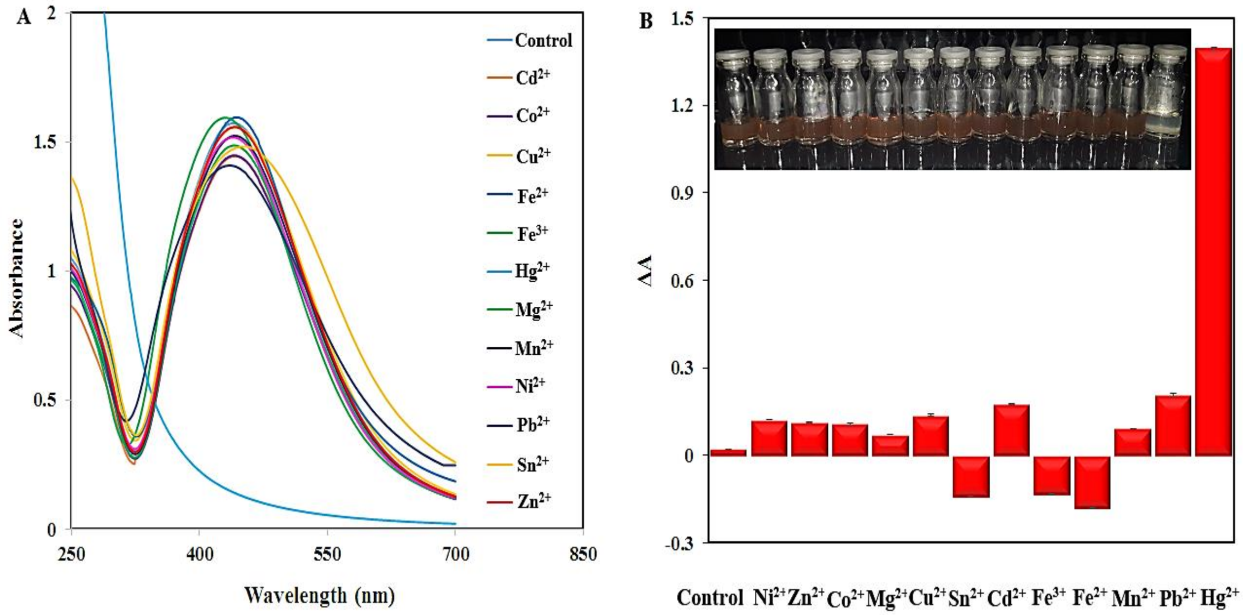

Figure S3: Selectivity observed for Aw-AgNPs sensing system toward Hg^{2+}. Absorption spectra of Aw-AgNPs solution after adding different metal ions (A); Colorimetric response of Aw-AgNPs solution to various metallic ions (B). Inset: the digital photographic images.

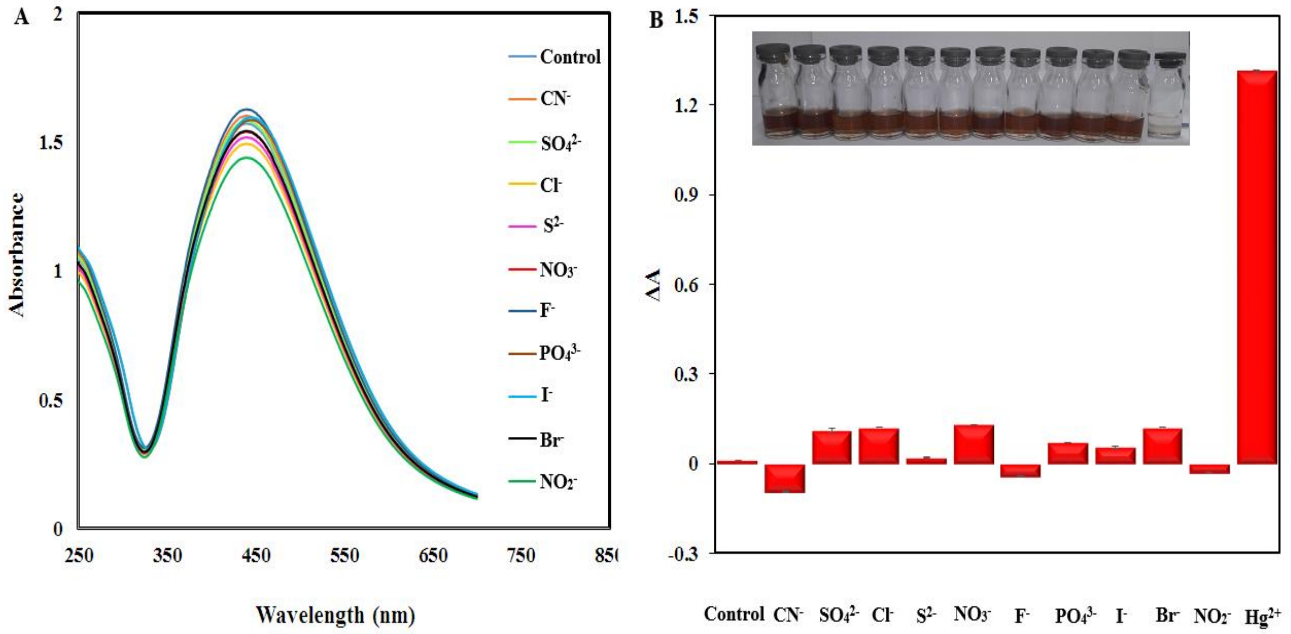

Figure S4: Selectivity observed for Aw-AgNPs sensing system toward Hg^{2+}. Absorption spectra of Aw-AgNPs solution after adding different anion ions (A); Colorimetric response of Aw-AgNPs solution to various anion ions (B). Inset: the digital photographic images.

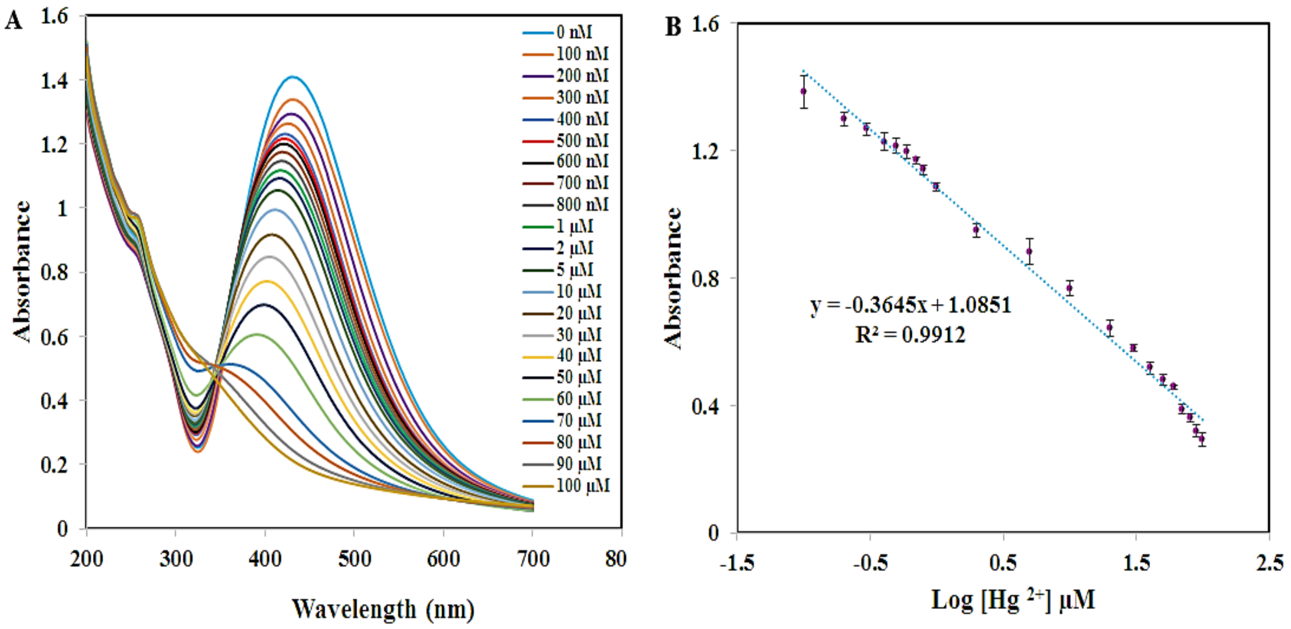

Figure S5: UV-Vis absorption spectra changes in in various concentrations of Hg^{2+} ions (response time= 5 min and range of analyte= 100 nM to 100 µM) (A); Hg^{2+} ions calibration curve plotted on a logarithmic scale (B).

Table S1. Recoveries of the Aw-AgNPs probe for colorimetric monitoring of Hg^{2+} ions in the real samples.

Sample	Amount added (µM)	Amount found (µM)	Recovery (%)	RSD (n = 3, %)
**Well water**				
1	20	22.1	110.5	1.87
2	50	53	106	2.37
3	100	94	94	3.92
**River water**				
1	20	18.1	90.5	2.93
2	50	53	106	3.64
3	100	97.2	97.2	1.32
**Lake water**				
1	20	21.5	107.5	2.80
2	50	54	108	4.61
3	100	101	101	3.29

References

[1] A. Chahardoli, F. Ghalekhani, A. Fattahi, Achillea wilhelmsii C. Koch mediated silver nanoparticles; a-blood compatible silver nanoparticles with potential application in wound healing.

[2] M. Mavaei, A. Chahardoli, Y. Shokoohinia, A. Khoshroo, A. Fattahi, one-step Synthesized Silver nanoparticles Using isoimperatorin: evaluation of photocatalytic, and electrochemical Activities, Scientific Reports, 10 (2020) 1-12.
